# Reinforcement of the Achilles Tendon Double-Row Repair: Technique Tip

**DOI:** 10.1177/24730114231182122

**Published:** 2023-06-28

**Authors:** Amanda Vandewint, Jacob Matz

**Affiliations:** 1 Faculty of Medicine, Dalhousie Medicine New Brunswick, Saint John, NB, Canada; 2 Canada East Spine Centre, Saint John, NB, Canada; 3 Division of Orthopaedic Surgery, Department of Surgery, Dalhousie University/DMNB, Saint John, NB, Canada

**Keywords:** Achilles tendon, insertional tendinopathy, intrasubstance suture construct, double-row fixation

## Introduction

Pathology of the Achilles tendon in the vicinity of its insertion onto the calcaneus may involve a combination of several entities. Insertional tendinopathy, referring to pathology where the Achilles inserts onto the calcaneus, is accompanied by tendon degeneration, as well as occasional bony spur formation. Achilles pathology slightly more proximal may be secondary to mechanical irritation from a Haglund’s deformity and accompanied by retrocalcaneal bursitis. When addressing these entities operatively, the typical process involves soft tissue and bony debridement, followed by tendon to bone reattachment and repair. Several different surgical repair techniques have been developed for this purpose.

Different repair options are compared from a biomechanical perspective by load-to-failure testing, as well as an anatomical perspective focused on the size of tendon to bone contact area. Prior studies have identified double-row constructs as performing superiorly in these areas in contrast to single-row fixation.^3,8^ Repair strength is crucial to allow the implementation of an early, aggressive course of postoperative rehabilitation while seeking to avoid complications of tendon detachment or elongation. Patient-reported outcomes related to quality of life at 1 year posttreatment and beyond have also been documented as significantly improved over preoperative scores following the use of a double-row suture fixation system.^5^ The weakness of this double-row arrangement is its reliance on very distal grasp of the tendon, leaving the possibility of the sutures pulling through the longitudinally oriented fibers of the Achilles.^4^

We present a novel modification to optimize the technique for performing a double-row repair at the Achilles insertion by employing a knotless intrasubstance suture construct with a Bunnell formation delivering a more substantial anchoring system.

## Technique

**Figure 1.**
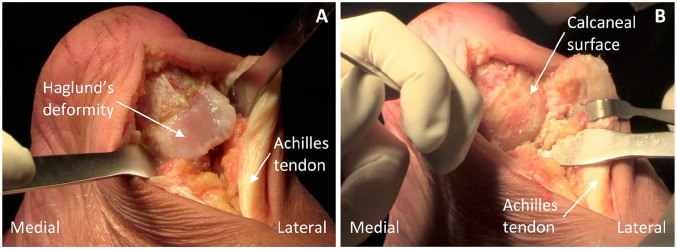
(A) View of the Haglund’s deformity with retraction of the overlying split medial and lateral Achilles tendon ends. (B) Calcaneal surface following Haglund’s deformity excision and subsequent bevelling of bony edges.

**Figure 2.**
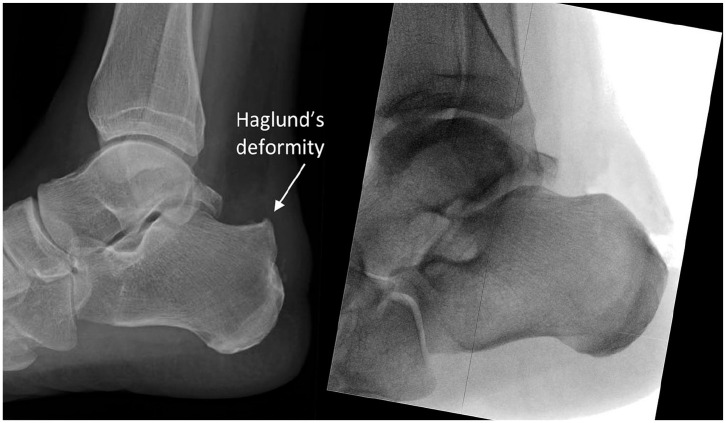
Comparison of preoperative lateral radiograph (left) showing Haglund’s deformity and intraoperative lateral radiograph (right) following bony debridement.

**Figure 3.**
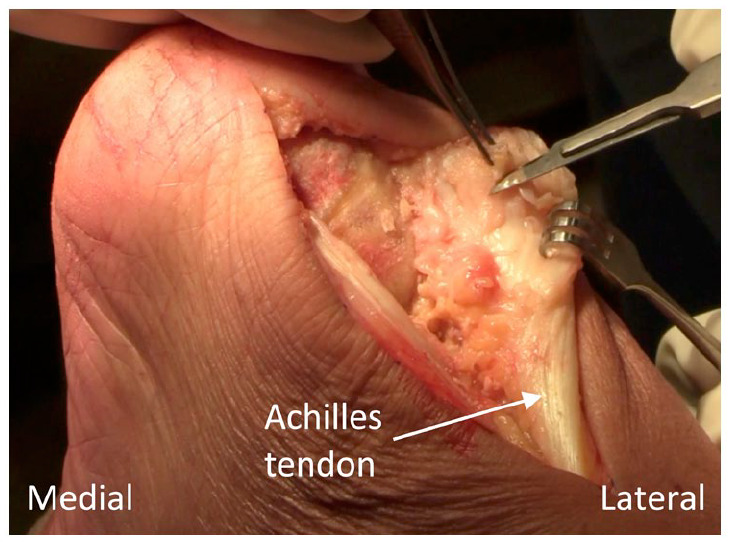
Careful debridement of the diseased portion of the Achilles tendon.

Anesthesia for the procedure consists of a general agent with case dependent addition of a nerve block. In prone positioning, the patient is secured on the operating table with their foot placed to hang over the lower table edge. Sterile prepping and draping of the patient’s leg is undertaken. Ischemia of the patient’s leg is achieved using a thigh tourniquet inflated to 300 mm Hg. Distal Achilles tendon debridement and Haglund’s deformity excision are carried out using a posterior central-splitting approach as previously described in the literature (Figures 1-3; Supplemental Video).^7^

**Figure 4.**
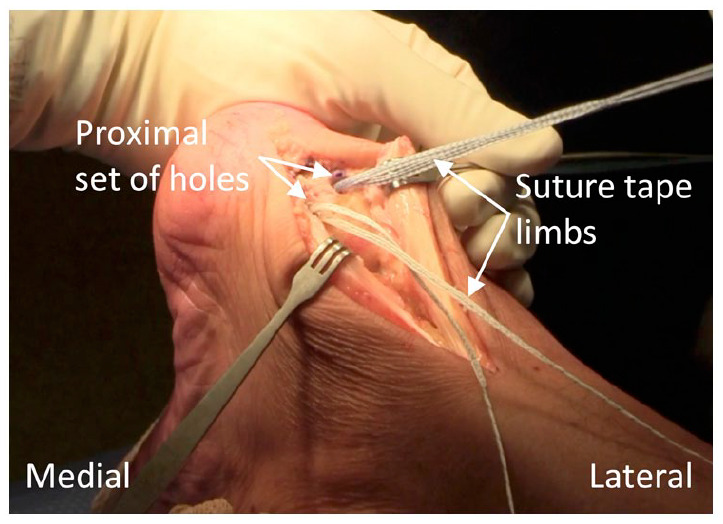
Position of the proximal set of holes each loaded with suture tape.

**Figure 5.**
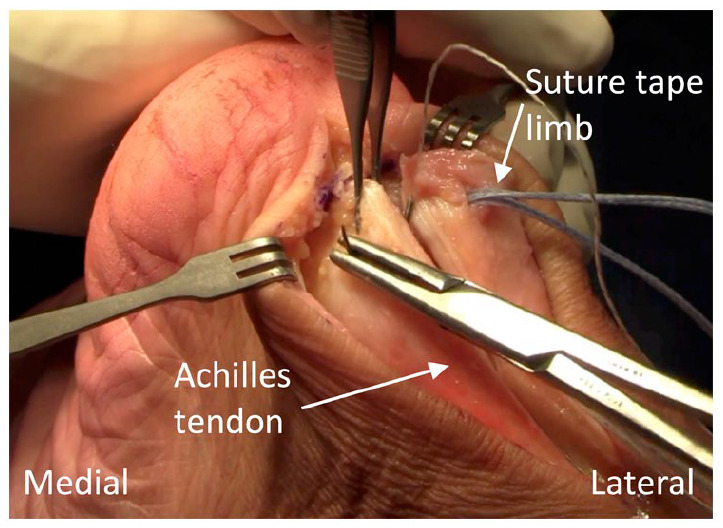
Passage of the suture tape from anterior to posterior through each tendon end.

Achilles reattachment follows using 4 anchors for a double-row configuration spanning the tendon insertion shown in this paper with the Achilles SpeedBridge Repair Implant System (Arthrex, Naples, FL) consisting of two 4.75-mm SwiveLock anchors loaded with No. 2 FiberWire and FiberTape sutures and two 4.75-mm SwiveLock anchors with No. 2 FiberWire suture alone.^2^ The proximal set of holes are first positioned approximately 1 cm proximal to the Achilles insertion point and at the midpoint of the tendon’s midline to lateral border distance for each split tendon end. The holes are drilled with a 3.5 mm drill bit and then prepared with an appropriately sized tap at which point the preloaded anchors are inserted (Figure 4). The suture tape is passed through the intrasubstance of each split tendon end (Figure 5). The distal set of holes are positioned and tapped in vertical alignment with the proximal holes, but just distal to the Achilles insertion point.

**Figure 6.**
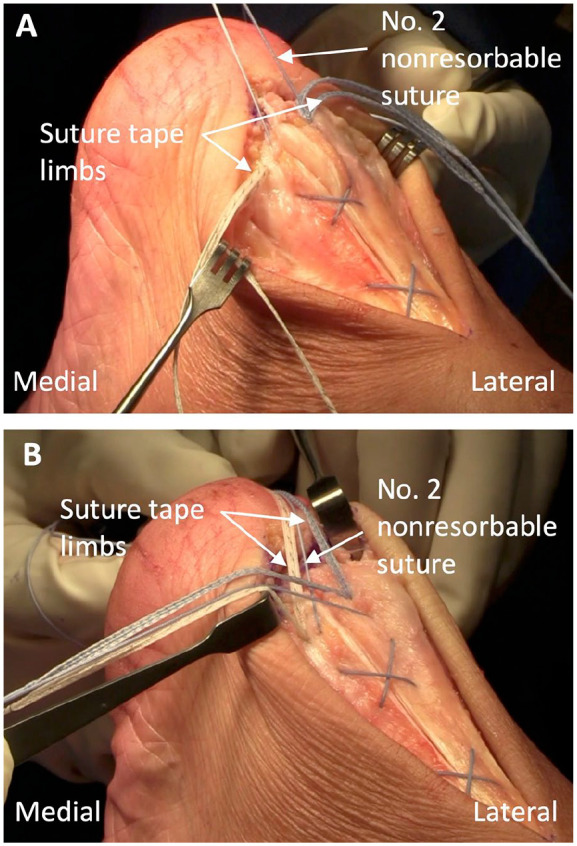
(A) Proximal to distal passage of an extra No. 2 nonresorbable suture through the Achilles tendon with most distal exit directed anterior to posterior. (B) Crossed arrangement of the suture tape limbs and the No. 2 nonresorbable suture ends.

**Figure 7.**
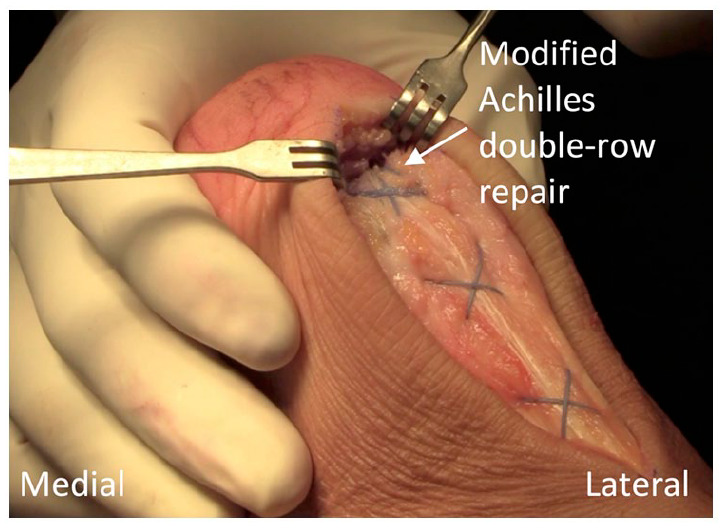
View of the modified double-row repair incorporating the additional No. 2 nonresorbable suture within the tendon intrasubstance in a Bunnell formation.

Before proceeding with anchor insertion for the distal holes, an extra No. 2 nonresorbable retention suture is passed through the tendon intrasubstance in a Bunnell formation from proximal to distal. The horizontal distance covered by each crossed section is approximated to match the distal 4-hole footprint. Two sections of suture crossing are performed with sufficient tension to reapproximate the tendon ends and with the most distal suture exit through the tendon in an anterior to posterior direction for incorporating the suture tails into the anchoring construct (Figure 6A). The remaining anchors are loaded with one suture tape from each proximal anchor, as well as the No. 2 nonresorbable retention suture positioned on the opposite side, and inserted into the appropriate holes with suitable tension to restore the footprint of the Achilles (Figures 6B and 7).

**Figure 8.**
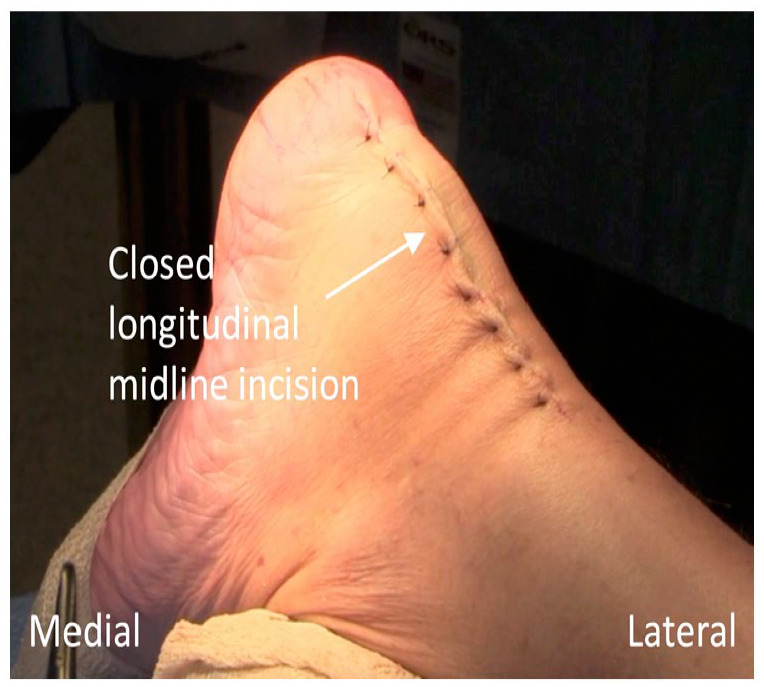
Closed appearance of the longitudinal midline incision.

Closure of the paratenon, subcutaneous, and cutaneous layers is undertaken after trimming excess suture limbs from the distal anchor sites (Figure 8). The postoperative protocol described by Akoh and DeOrio^1^ is followed by placing the foot in a splint with no weightbearing for 3 weeks initially and then transitioning to a walking boot for progressive increases in levels of weightbearing and attempted range of motion.

## Discussion

The repair strength conferred by Achilles tendon reattachment using double-row fixation provides clear evidence in support of using such a technique compared to available alternatives.^3,8^ However, in the event of the proximal suture pulling through the longitudinally oriented Achilles tendon fibers, the overall construct would be compromised.^4^ Augmenting this technique with additional safeguards is desirable. Hoffman et al^6^ have described such a failsafe in the form of incorporation of a simple rip-stop suture providing another layer of support to the repair. The use of the preloaded suture from the medial-distal anchor passed in a running manner distally to proximally within the Achilles tendon intrasubstance has also been described by Akoh and DeOrio^1^ as a means of reapproximating the split ends of the tendon.

The modification we are presenting employs a No. 2 nonresorbable suture in a Bunnell formation, passed through the tendon in the proximal to distal direction and anchored within the distal row of the double-row construct. This knotless reinforcement allows the surgeon to tension the repair appropriately without fear of the suture fibers cutting through the distal Achilles tendon.

## Supplemental Material

sj-pdf-1-fao-10.1177_24730114231182122 – Supplemental material for Reinforcement of the Achilles Tendon Double-Row Repair: Technique Tip Click here for additional data file. Supplemental material, sj-pdf-1-fao-10.1177_24730114231182122 for Reinforcement of the Achilles Tendon Double-Row Repair: Technique Tip by Amanda Vandewint and Jacob Matz in Foot & Ankle Orthopaedics
